# Addressing Racial and Ethnic Disparities in COVID-19 Among School-Aged Children: Are We Doing Enough?

**DOI:** 10.5888/pcd18.210084

**Published:** 2021-06-03

**Authors:** Arica White, Leandris C. Liburd, Fátima Coronado

**Affiliations:** 1 Division of Cancer Prevention and Control, Centers for Disease Control and Prevention, Atlanta, Georgia; 2 COVID-19 Response, Chief Health Equity Officer, Centers for Disease Control and Prevention, Atlanta, Georgia; 3 Division for Heart Disease and Stroke Prevention, Centers for Disease Control and Prevention, Atlanta, Georgia

## Abstract

The disproportionate impact of COVID-19 and associated disparities among Hispanic, non-Hispanic Black, and non-Hispanic American Indian/Alaska Native children and teenagers has been documented. Reducing these disparities along with overcoming unintended negative consequences of the pandemic, such as the disruption of in-person schooling, calls for broad community-based collaborations and nuanced approaches. Based on national survey data, children from some racial and ethnic minority groups have a higher prevalence of obesity, asthma, type 2 diabetes, and hypertension; were diagnosed more frequently with COVID-19; and had more severe outcomes compared with their non-Hispanic White (NHW) counterparts. Furthermore, a higher proportion of children from some racial and ethnic minority groups lived in families with incomes less than 200% of the federal poverty level or in households lacking secure employment compared with NHW children. Children from some racial and ethnic minority groups were also more likely to attend school via online learning compared with NHW counterparts. Because the root causes of these disparities are complex and multifactorial, an organized community-based approach is needed to achieve greater proactive and sustained collaborations between local health departments, local school systems, and other public and private organizations to pursue health equity. This article provides a summary of potential community-based health promotion strategies to address racial and ethnic disparities in COVID-19 outcomes and educational inequities among children and teens, specifically in the implementation of strategic partnerships, including initial collective work, outcomes-based activities, and communication. These collaborations can facilitate policy, systems, and environmental changes in school systems that support emergency preparedness, recovery, and resilience when faced with public health crises.

**Summary**

**What is already known on this topic?**

The disproportionate impact of COVID-19 and associated disparities among some racial and ethnic minority populations has been documented across age groups, including children and teens.

**What is added by this report?**

We provide a summary of potential community-based strategies that can be used to address health disparities and educational inequities among minority children and teens that have been exacerbated during the pandemic.

**What are the implications for public health practice?**

Evidence-based community health promotion models that center partnerships in a strategic and comprehensive approach may reduce racial and ethnic health disparities and educational inequities due to COVID-19 and advance health equity.

## Introduction

The population health impact of COVID-19 has exposed decades, if not centuries, of inequities that have systematically undermined the physical, social, material, and emotional health of racial and ethnic minority groups (1,2). The disproportionate impact of COVID-19 and associated disparities in outcomes among some racial and ethnic minority populations is documented across age groups, including among children (3–5). Reducing these disparities along with the inequitable economic and social impact of the pandemic on families from racial and ethnic minority groups requires broad community-based and underused collaborations, as well as innovative approaches.

In this article, we highlight health disparities and inequities among children and teenagers from racial and ethnic minority groups. We discuss education as a major social determinant of health and the impact of restricted access to in-person school, and we describe disparities in underlying chronic medical conditions and social inequities associated with poverty and systemic disadvantage. In combination, these factors exacerbate poor health outcomes in populations disproportionately affected by social conditions beyond their control, including infection with severe acute respiratory syndrome coronavirus 2 (SARS-CoV-2), the virus that causes COVID-19. In light of documented disparities and the potential collateral damage inflicted by the COVID-19 pandemic, such as delays in health care, increases in social isolation, and decreases in vaccination rates (6), we invoke a renewed sense of urgency in attending to the population health status of children and teens from racial and ethnic minority groups.

We argue for community-based approaches that are organized to achieve greater proactive and sustained collaborations between local health departments, local school systems, and other public and private organizations. Although these approaches are not new, the impact of the COVID-19 pandemic on school-aged children experiencing systemic disadvantage reintroduces and amplifies the need for community-based collaborations and strategic partnerships. Since the causes of health disparities are complex and multifactorial, eliminating these health disparities cannot be accomplished by a single sector or entity (7). As trusted members of their communities, partners from the public and private sectors can play a key role in improving population health (8). Revitalizing old partnerships and establishing new collaborations may reduce COVID-19 disparities; improve and protect the physical and mental health of children and teens from racial and ethnic minority groups experiencing systemic disadvantage; and advance health equity, which is the opportunity for everyone to be as healthy as possible (2). We posit that these partnerships and collaborations can facilitate policy, systems, and environmental changes within school systems that support emergency preparedness and recovery and resilience when faced with public health crises.

## Education is a Major Social Determinant of Health

Education is a major social determinant of health and is essential to achieving health equity (9). Educational attainment and disparities in health are closely linked (10). Moreover, education is highly correlated with income and occupation, and “less education predicts earlier death” (11). Furthermore, through occupational attainment, education most often determines access to health care and health-related benefits, including paid time off and paid sick leave (12). Adults with less education report worse general health, more chronic conditions, and more functional limitations than those with higher levels of education (13,14).

School closures (during the spring of 2020) in response to the COVID-19 pandemic, including in-person kindergarten through grade 12 (K-12) schools, and the safe reopening of schools and institutions of higher education have been at the center of public health decision making to ensure guidelines protect students, teachers, faculty, and staff. According to Fay et al, along with the economy and the health care system, schools are “a third pillar of a pandemic-resilient society” (15). Besides education, schools provide facility-based services on which many students and families rely, including academic intervention supports, food and nutrition programs, childcare, after-school support, and social, physical, and mental health services. Schools can also serve as an extension of the home environment and offer a protective social environment for some students (16). As enduring community institutions, services provided by schools to “communities made vulnerable by systemic racism, inadequate insurance, family instability, environmental toxicity, and poorly paid jobs” (17) are essential to the overall well-being and psychosocial health of students experiencing poverty and systemic disadvantage (12). In this article, students experiencing systemic disadvantage include those who are disproportionately from racial and ethnic minority groups, with disabilities, experiencing homelessness, in foster care, and for whom English is a second language (18).

Children who experience poverty and systemic disadvantage, who are more likely to be from racial or ethnic minority groups, may be at higher risk of infection, severe illness, and death from COVID-19 (4). Because of school closures, children already experiencing disadvantage may have limited access to the facility-based services, academic supports (eg, private instructors, learning pods), virtual learning options, and digital technologies needed to successfully complete their grade-level academic requirements (18). Parents’ lives are also impacted by school closures. Some parents may not be able to return to work or may not have paid leave, flexible schedules, or options to work remotely and may have to balance how they provide supervision to several children at home with different academic schedules (17,18). Parents’ comfort with their own educational attainment and confidence to help their children academically is also a factor in how well students perform (19,20). These challenges may widen the gap in academic performance for students experiencing disadvantage compared with their more privileged counterparts. Conversely, in-person learning during the pandemic may pose challenges for these children and their families. Schools that serve communities with a disproportionate number of people experiencing poverty are often under-resourced, overcrowded, and understaffed, which increases risk for COVID-19 transmission in schools and adds to the challenges associated with safe reopening (17). Furthermore, some students may live with others who, for various reasons, are at increased risk for COVID-19 infection or live in intergenerational or crowded housing, which may influence their parent’s or guardian’s decision to send them to in-person school. Under-resourced schools may also have reduced capacity to offer high-quality virtual learning or be able to provide the supports needed for students with disabilities or other special needs. To mitigate the impacts of COVID-19 on the scope and quality of educational resources available to these students, decision makers, programs, and interventions must consider health disparities and social inequities and act on the unique conditions that could increase students’ risk of infection and severe illness from COVID-19.

## Disparities in Underlying Medical Conditions and Social Determinants of Health

As of May 2021, the US continues to experience substantial levels of SARS-CoV-2 transmission. Although less common than in adults, children and teens are still at risk of developing severe illness and complications from COVID-19; approximately 1 in 3 children hospitalized with COVID-19 were admitted to the intensive care unit, similar to the rate among adults (4). Although evidence on which medical conditions in children are associated with increased risk is limited, children with the following conditions might be at increased risk for severe COVID-19: obesity, diabetes, asthma, other chronic lung disease, congenital heart disease, medical complexity, severe genetic disorders, sickle cell disease, chronic kidney disease, severe neurologic disorders, inherited metabolic disorders, and immunosuppression due to malignancy or immune-weakening medications (21).

Serious racial and ethnic health and health care inequities persist for children with chronic health conditions (22). National estimates indicate significant disparities in the prevalence of chronic disease conditions that may place some children and teens at increased risk for severe illness from COVID-19 (Table 1). Nearly 1 in 5 children aged 2–19 years (19.3%) in the United States have obesity (24). The prevalence of obesity among Mexican American (26.9%), Hispanic (25.6%), and non-Hispanic Black (NHB; 24.2%) children was higher than among non-Hispanic White (NHW; 16.1%) and non-Hispanic Asian (8.7%) children during 2017-2018. Children who have obesity are more likely to have risk factors for adult cardiovascular disease (27), including high blood pressure and high cholesterol, increased risk of impaired glucose tolerance, insulin resistance, and type 2 diabetes, as well as asthma (28) and sleep apnea (29). Racial and ethnic disparities also are evident in asthma, diabetes, and cardiovascular disease. In 2018, the prevalence of current asthma among NHB (14.2%), Hispanic overall (8.0%), and Mexican American (7.0%) children was higher than among NHW (5.6%) children younger than 18 years (Table 1).

**Table 1:** Estimates of Selected Chronic Conditions Among Children and Teenagers, by Race/Ethnicity, United States

Chronic Condition (Age, y)	Estimate
**Current asthma (<18)^a^**	**Age-Adjusted Prevalence, % (SE)**
White, non-Hispanic	5.6 (0.40)
Black, non-Hispanic	14.2 (1.52)
Hispanic	8.0 (0.85)
Mexican American	7.0 (1.01)
**Obesity (2-19)^b^**	**Prevalence, % (SE)**
White, non-Hispanic	16.1 (1.5)
Black, non-Hispanic	24.2 (2.0)
Hispanic	25.6 (1.9)
Mexican American	26.9 (2.5)
Asian, non-Hispanic	8.7 (1.1)
**Type 2 diabetes (10–19)^c^**	**Incidence Rate Per 100,000**
White, non-Hispanic	4.5
Black, non-Hispanic	37.8
Hispanic	20.9
American Indian	32.8
Asian/Pacific Islander	11.9
**Hypertension (12–19)^d^**	**Prevalence, % (95% CI)**
White, non-Hispanic	2.97 (1.73–4.74)
Black, non-Hispanic	6.27 (3.84–9.59)
Mexican American	4.94 (3.01–7.59)
Other	5.22 (3.65–7.20)

a 2018 data from the National Health Interview Survey (23).

b 2017–2018 data from the National Health and Nutrition Examination Survey (24).

c 2014–2015 data from the SEARCH for Diabetes in Youth Study (25).

d 2013–2016 data from the National Health and Nutrition Examination Survey (26).

The SEARCH for Diabetes in Youth Study has reported disparities in the incidence of type 2 diabetes per 100,00 among children aged 10–19 years. During 2014–2015, NHB (37.8), American Indian (32.8), Hispanic (20.9), and Asian/Pacific Islander (11.9) children had higher incidence rates of type 2 diabetes than NHW (4.5) children (Table 1) (25). Additionally, using 2013–2016 data from the National Health and Nutrition Examination Survey (NHANES), Jackson et al reported that among children aged 12–19 years, the estimated prevalence of hypertension (≥130/80 mm Hg) was 4.2%. However, the prevalence for NHB (6.3%) and Mexican American (4.9%) children was higher than among NHW children (3.0%) (26). According to Lopez et al, mortality rates resulting from congenital heart disease significantly declined during 1999–2017 among all races/ethnicities, although disparities in mortality rates persisted among NHB children in comparison with NHW children; the highest mortality rate was in infants (<1 year) of all races/ethnicities (30). Improvements in cardiovascular health have not been equally shared by US children aged 12–19 years of varying socioeconomic status. A study using NHANES data reported increases in the prevalence of obesity among only adolescents from low-income (18.1%–21.7%) and middle-income (17.1%–26.0%) households from 1999 to 2014. During 2011–2014, significant disparities in prevalence of obesity were observed between adolescents from low-income and high-income households (21.7% vs 14.6%). Although no significant disparities were observed in children aged 12–19 years in the prevalence of prediabetes, diabetes, hypertension, or hypercholesterolemia, the prevalence of prediabetes and diabetes increased (21.4%–28.0%) among adolescents from low-income households during 1999–2014 (31).

Approximately 42% of children hospitalized with COVID-19 during March 1 through July 25, 2020, had 1 or more underlying medical conditions (4). The most prevalent conditions among these children were obesity (37.8%) and chronic lung disease, including asthma (18.0%). For hospitalized children aged 5–17 years, Hispanic (42.3%) and NHB (32.4%) children had a higher prevalence of underlying conditions compared with NHW children (14.1%); Hispanic (47.2%) and NHB (31.8%) children also had higher hospitalization rates than NHW children (12.6%) (4).

The families of children experiencing systemic disadvantage likely share similar COVID-associated health risks and, therefore, may be more likely to be hospitalized or die if they contract COVID-19 (2,32). As of November 30, 2020, compared with NHW individuals, hospitalization rate ratios were 4 times higher among non-Hispanic American Indian or Alaska Native people and Hispanic or Latino people, and 3.7 times higher among NHB or African American people (32). Likewise, deaths were 2.8 times higher for NHB or African American people and Hispanic or Latino people, and 2.6 times higher for non-Hispanic American Indian or Alaska Native people compared with NHW people (32). Family and household members may be at increased risk of exposure to COVID-19 through their occupation (33). Parents then play an important role in ensuring strict adherence to established mitigation measures by everyone in the household (34).

## Inequities in Social Determinants of Health 

Racial or ethnic minority populations are more likely to experience lower socioeconomic status, live in crowded housing, and possibly be employed in occupations that require in-person work (2). Furthermore, access to health care may be limited, including obtaining testing and care for COVID-19 (2). Compared with NHW (26%) and Asian/Pacific Islander children (25%), a larger proportion of NHB (58%), American Indian (56%), and Hispanic (53%) children younger than 18 years lived in families with incomes less than 200% of the federal poverty level in 2019 (35). Compared with NHW (21%) and Asian/Pacific Islander children (21%), a larger proportion of NHB (41%), American Indian (44%), and Hispanic (31%) children’s parents lack secure employment (35). In addition, Hispanic and NHB children, regardless of their families’ income, are more likely than NHW or Asian children to attend schools with a high proportion of students from families with incomes below the federal poverty level (36).

School districts that serve a high proportion of students who are from racial and ethnic minority populations and students who are from families with lower incomes receive less state and local funding than schools that serve a lower proportion of these groups (37). School funding determines the availability of student supports, classroom sizes, and a myriad of other factors that can affect student learning (37). Under-resourced schools may be unable to sufficiently address students’ academic, social, emotional, and mental health needs that were exacerbated by the COVID-19 pandemic without support from community institutions and resources, including public health. However, in light of new federal funding through the American Rescue Plan (38), these school districts have a new opportunity to invest in meaningful and productive partnerships.

In addition to the potential for overcoming educational inequities, promotion of resilience may prevent or ameliorate the impacts of social adversity on children. Evidence suggests that specific individual (eg, cognitive skills, emotion regulation, self-esteem), relational (eg, relationships with caregivers), and school factors (eg, academic engagement) are associated with resilience (39). Factors that promote resilience can be considered at multiple levels (eg, individual, family, environmental) and are complimentary to public health efforts (40).

## Community-Based Approaches to Reducing COVID-19 Disparities

Understanding the social context of populations with high rates of COVID-19 infection and severe illness is critical to the development, implementation, and evaluation of public health prevention strategies. Although structural long-term solutions to eliminating racial and ethnic health disparities are optimal and preferred (41), evidence suggests that immediate relief and support during the COVID-19 pandemic can be achieved when local public health departments, school leaders, and community partners join forces. For example, the Coordinated Approach to Child Health: Curriculum & Training (CATCH) program consists of comprehensive and coordinated programs, policies, and services that involve partnerships between families, schools, and the community (42). This school health program focuses on coordinating the efforts of teachers, school staff, and the community to promote healthy behaviors to prevent childhood obesity. Through this approach, programs had greater impact in reducing overweight and obesity when schools worked with community-based partners (42). Using a coordinated approach can impact the way communities conceptualize and address problems and can enhance implementation of strategies (8). This approach may help address the unique challenges some children face throughout the pandemic and support transitions into early pandemic recovery and beyond.

Another way to inform focused prevention strategies is for school districts to develop plans that can be tailored at the individual school level to address gaps in learning and well-being for the students. According to a study by researchers at Johns Hopkins University, most state and territorial boards of education (89%, 48 of 54) have individual plans with provisions to narrow gaps in learning and well-being that may have been exacerbated by school closures for children experiencing poverty and systemic disadvantage (43). Some of these provisions include providing access to digital technologies and corresponding training and support for students and parents; special virtual instructional support (eg, tutoring); prioritization of children experiencing disadvantage for in-class instruction; and accommodation of schedule-related or childcare needs of parents with lower income, people of color, or essential workers. Because states and school districts may have implemented their reopening plans differently, partnerships and collaboration with public health departments and community-based organizations could help with monitoring the execution and reach of those plans as well as assessing critical needs to ensure that equity considerations are implemented. Examining these provisions can inform models and standards to use during the COVID-19 pandemic and for emergency preparedness planning.

Plans should be comprehensive and consider disparities in conditions that could affect educational achievement, including mental health and emotional well-being, within the context of the COVID-19 pandemic. For example, compared with 2019, the proportion of mental health–related visits for children aged 5–11 and 12–17 years increased approximately 24% and 31%, respectively; these increases began in April 2020, corresponding to the time in which many schools were required to close (44). Younger adults (aged 18–24 y), Hispanic people, NHB people, essential workers, and unpaid caregivers for adults reported having experienced disproportionately more adverse mental health outcomes (45).

Zimmerman et al found what they describe as “nuanced contextual covariables in our society that provide a fuller back story” to the complex association between educational attainment and health outcomes (46). Namely, they identified social skills, emotional dysregulation, trauma, abuse, and neglect, among other variables that should be addressed when the goal is to increase educational attainment. Moreover, Hahn and Truman argue that another essential element in the pathway from educational attainment to health outcomes is the “psychosocial environment,” which includes sense of control (eg, work-related factors, health-related behaviors, stress), social standing (social and economic resources, stress), and social support (social and economic resources, health behaviors, family stability, stress) (10). If these variables require attention absent a global pandemic, then they cannot be ignored during this public health crisis. Partnerships can facilitate obtaining resources to promote coping and resilience, reduce health and mental health disparities, and expand access to services to support children’s and teens’ mental health. For example, schools could help link children and their families to community health centers for affordable mental health support services.

## Implementing Strategies to Advance Health Equity Through Partnerships

Community-based public and private sector partnerships are a cornerstone of community health promotion, chronic disease prevention, and a range of health equity initiatives. In addressing COVID-19 disparities and consequent social and health inequities, we borrow from the evidence base and experience of other public health interventions. Dicent Taillepierre and colleagues identified several elements in program design that enhance health equity, including consideration of sociodemographic characteristics, understanding the evidence base for reducing health disparities, leveraging multisectoral collaboration, using clustered interventions, engaging communities, and conducting rigorous planning and evaluation (47). Considering these elements and other experiences that support the benefit of community-based partnerships, we propose immediate actions that can be taken to respond to the pandemic, as well as to establish and track outcomes (34,47).

We propose 4 evidence-based approaches to form community-based partnerships, including initial collective work, outcome-based activities, and communication efforts, that collaborators can use to improve health equity among students from racial and ethnic minority groups (Table 2). First, education departments should identify organizations with the mission and expertise to support tailored efforts to ameliorate education inequities among children and teens who are experiencing systemic disadvantage and falling behind academically. Multiple sectors and community actors such as clergy and faith-based organizations, YMCA, YWCA, Boys & Girls Clubs of America, Head Start programs, federally qualified community health centers, and parent–teacher associations can be effective community-based partners to protect students and support access to equitable education (Table 2).

**Table 2:** Summary of Potential Community-Based Strategies to Address Racial and Ethnic Disparities in COVID-19 Outcomes Among Children and Teens

Partnerships: public health and boards of education work together with cross-sector partners^a^
• Identify and engage community partners with interest in or established relations working with K-12 schools serving minority populations • Engage women’s, parents’, and adolescent and youth groups to ensure there is effective peer outreach • Leverage trusted community resources, influencers, and other community leaders (eg, faith-based organizations) and businesses (eg, Adopt a School Program) • Facilitate innovative partnerships to include auxiliary services (eg, Head Start programs, YMCA, YWCA, Boys and Girls Club of America, cooperative extension services) • Include other governmental (eg, local parks and recreations departments) and nongovernmental organizations (eg, Food Bank programs) • Develop partnerships with a priority based on equity and removing systemic barriers of students experiencing disadvantage • Incorporate community oversight of activities to ensure their voice is represented and to build trust in the community • Gather, share, and use data (race, ethnicity, language, location, social factors) to focus efforts
**Initial collective work: partnerships collectively define the problem and create a shared vision to solve it^b^**
• Plan community-based support for learning, families, and whole child development • Conduct rapid assessment of the digital literacy capacity for students and teachers • Conduct rapid assessment of barriers to virtual learning • Identify resources needed by students to receive optimal virtual learning • Conduct rapid assessment of barriers to safe (return to) in-person learning • Collaborate to identify and fill the gaps in mitigation efforts in schools • Support under-resourced schools in planning to mitigate exposure and transmission of COVID-19, as well as testing and contact tracing • Identify other health-related needs
**Outcomes-based activities: partnership leads and/or facilitates activities with a focus on outcomes^c^**
• Address barriers to optimal virtual learning (eg, limited access to technology and eLearning materials, experience, instructor issues) • Quantify tutoring and mentoring needs and invest in rapid, remote support (eg, local volunteers tutor remotely) • Address barriers to in-person learning (eg, implementation of mitigation strategies in K-12 schools, safe transportation) • Pilot test and evaluate mitigation strategies among K-12 schools that serve a high proportion of students from racial and ethnic minority populations or from families with low incomes or limited resources • Support the capacity to identify and respond to signs of stress, isolation, or poor mental health in students • Protect students from abuse/violence • Improve adolescents’ health literacy related to the COVID-19 pandemic and general health • Identify and facilitate afterschool and expanded learning opportunities
**Communications: leverage existing and innovative approaches using focused messages^d^**
• Use existing digital platforms for small group or individual tele-tutoring (eg, 2 times per week) to strengthen learning, social engagement, and cultural affirmation • Schools identify and address inclusive delivery mechanisms for students with disabilities who are learning remotely • Develop and deliver accurate and culturally responsive information about COVID-19 and how students can protect themselves (eg, ensure language access, broad distribution through trusted sources, and relevance to students from racial or ethnic minorities) • Provide teleconsultations/telecounseling and educational sessions (webinars) to families about coping with stress and providing information on positive parenting • Provide resources and links to psychosocial and mental health support services (eg, community health centers and other health-related social needs) • Implement messages to increase awareness and resources for children and teens experiencing abuse/violence • Provide outreach activities and culturally tailored prevention messages/campaigns focused on COVID-19 • Provide outreach activities and culturally tailored prevention messages focused on preventing chronic diseases (eg, healthy eating, exercise)

a Hann NE (48).

b Dankwa-Mullan I and Perez-Stable EJ (49).

c Porterfield DS et al (50).

d Bernhardt JM (51).

Relevant community partners can supplement available resources and sponsor critical activities to meet students’ unique needs (48). Participating community-based organizations should be aware of the characteristics of a community, including language, race, ethnicity, countries of origin, and other factors that could affect health status, access to health care, and the provision of culturally and linguistically responsive prevention messages (52).

Second, to facilitate successful collaborations, initial collective work by partners is needed to define the problem and create a shared vision to achieve specific outcomes. Assessments to inform policy, systems, and environmental change are needed. These assessments can include public health data describing the impact of COVID-19 in the community of interest, particularly among children and teens enrolled in school; school system equity plans to mitigate exposure and transmission of COVID-19; and reviews of the school system’s digital learning capacities. Place-based approaches can align community members, businesses, institutions, and others in a collaborative and participatory process to address health and contextual factors influencing the social well-being of children within a defined community (49). For example, these efforts planned with community members offer an opportunity to strategically assess and monitor trends in population health status and the needs and assets of a community.

Third, it is important for partners to take the lead or facilitate activities that focus on outcomes they have the capacity to achieve. For example, the Boys & Girls Club of America can provide tutoring services and other extra-curricular activities to minimize academic delays and poor performance on standardized tests. Later, rigorous program evaluations can document the effectiveness of these strategies post-pandemic (50).

Finally, communication is one of the core components for promoting and improving public health (51). Ongoing communication between schools, parents, and community-based organizations is essential. Particularly, a commitment to transparency is needed so that parents and the larger community are kept apprised of partnership efforts and informed when outcomes are on track. Partners can leverage various media outlets, including social media, to disseminate tailored prevention messages as well as connect students and parents to health care services. For example, existing digital platforms can be used for tutoring small groups or individual students. Telemedicine, including telehealth technologies, can be used to provide counseling to families about coping with stress. Although these evidence-based approaches are not new to public health, there are new opportunities to scale these approaches for greater reach and impact in communities disproportionately impacted by COVID-19.

Because of their critical role for all children and the disproportionate impact that school closures can have on those students experiencing systemic disadvantage, it is crucial that K-12 schools open safely and remain open for in-person learning (53). Community engagement and partnerships are foundational to public health and its core value of social justice (54). Partnerships can help facilitate delivery of quality virtual learning, policies, and systems changes that keep classrooms safe for in-person learning, and they can facilitate communication strategies that ensure the dissemination of scientifically sound public health prevention strategies that build community confidence in the safe reopening of schools. In addition to facilitating and sustaining in-person learning, partnerships can help prevent further exacerbation of educational inequities, support parents’ full return to work and more everyday activities in different settings, and fuel economic recovery. Because the needs, risk factors, assets, and resources vary across communities, local public health departments and school boards of education should work with local organizations that can help provide tailored support. Moreover, local organizations are more likely to be perceived as trustworthy and credible by communities (52). Recent federal funding opportunities can help facilitate and sustain these partnerships. The American Rescue Plan Elementary and Secondary School Emergency Relief Fund, with funds totaling $122 billion, supports efforts by states, Puerto Rico, and the District of Columbia to reopen K-12 schools safely and to equitably expand opportunity for students experiencing disadvantage (38). These funds can be used to implement strategies, including evidence-based interventions, to meet the social, emotional, mental health, and academic needs of students. Furthermore, the Centers for Disease Control and Prevention (CDC) is providing $10 billion to states to support COVID-19 screening and testing for K-12 teachers, staff, and students (38). Partnerships can leverage these funding opportunities and aid the implementation of rapid response efforts needed to facilitate learning.

The COVID-19 pandemic has not only exposed longstanding health and social inequities in the US but also revitalized efforts to achieve authentic community engagement in promoting mitigation efforts to end the pandemic. Partnerships between local health departments, local school systems, and other public and private organizations can offer immediate support to these children and teens during the COVID-19 pandemic and over the long term as we move into the recovery phase.
